# Supplementary material for: Oxidation of fish oil exacerbates alcoholic liver disease by enhancing intestinal dysbiosis in mice
Source: Commun Biol. 2020 Sep 2;3:481. doi: 10.1038/s42003-020-01213-8 (PMC7468239; doi:10.1038/s42003-020-01213-8)
Supplement: Supplementary file 1 — Reporting Summary [file 42003_2020_1213_MOESM1_ESM.pdf]

nature research

Corresponding author(s): Jianbo Wan

Last updated by author(s): Jul 31, 2020

Reporting Summary

Nature Research wishes to improve the reproducibility of the work that we publish. This form provides structure for consistency and transparency in reporting. For further information on Nature Research policies, see Authors & Referees and the Editorial Policy Checklist .

Statistics

For all statistical analyses, confirm that the following items are present in the figure legend, table legend, main text, or Methods section.

n/a	Confirmed	
☐	☒	The exact sample size (*n*) for each experimental group/condition, given as a discrete number and unit of measurement
☐	☒	A statement on whether measurements were taken from distinct samples or whether the same sample was measured repeatedly
☐	☒	The statistical test(s) used AND whether they are one- or two-sided<br>*Only common tests should be described solely by name; describe more complex techniques in the Methods section.*
☒	☐	A description of all covariates tested
☐	☒	A description of any assumptions or corrections, such as tests of normality and adjustment for multiple comparisons
☐	☒	A full description of the statistical parameters including central tendency (e.g. means) or other basic estimates (e.g. regression coefficient) AND variation (e.g. standard deviation) or associated estimates of uncertainty (e.g. confidence intervals)
☐	☒	For null hypothesis testing, the test statistic (e.g. *F*, *t*, *r*) with confidence intervals, effect sizes, degrees of freedom and *P* value noted<br>*Give P values as exact values whenever suitable.*
☒	☐	For Bayesian analysis, information on the choice of priors and Markov chain Monte Carlo settings
☒	☐	For hierarchical and complex designs, identification of the appropriate level for tests and full reporting of outcomes
☒	☐	Estimates of effect sizes (e.g. Cohen's *d*, Pearson's *r*), indicating how they were calculated

Our web collection on statistics for biologists contains articles on many of the points above.

Software and code

Policy information about availability of computer code

Data collection

1. Oxidative parameters (e.g. POV, Totox, and TBARS) were determined using UV-1800-UV-IVS Spectrophotometer
2. Real-time PCR was performed using Mx3005P Q-PCR system
3. Fatty acid profiles were performed using gas chromatography–mass spectrometry (GC-MS) equipped with a TriPlus RSHTM autosampler (Thermo Fisher Scientific, Waltham, MA, USA).
3. Nuclear p65 expression was analyzed using laser-scanning microscope Leica TCS SP8
4. Immunofluorescence assay was captured using an inverted fluorescence microscope (IX83, Olympus, Japan)
5. Biochemical parameters were performed using a SpectraMax M5 microplate reader
6. Western blot bands were analyzed using Bio-Rad ChemiDoc™ XRS System

Data analysis

Statistical analysis was performed using GraphPad Prism 6.0 software.

For manuscripts utilizing custom algorithms or software that are central to the research but not yet described in published literature, software must be made available to editors/reviewers. We strongly encourage code deposition in a community repository (e.g. GitHub). See the Nature Research guidelines for submitting code & software for further information.

Data

Policy information about availability of data

All manuscripts must include a data availability statement. This statement should provide the following information, where applicable:

- Accession codes, unique identifiers, or web links for publicly available datasets
- A list of figures that have associated raw data
- A description of any restrictions on data availability

Raw data underlying plots in figures are available in Supplementary Data 1. Full blots are provided in Supplementary Information.

Field-specific reporting

Please select the one below that is the best fit for your research. If you are not sure, read the appropriate sections before making your selection.

☒ Life sciences ☐ Behavioural & social sciences ☐ Ecological, evolutionary & environmental sciences

For a reference copy of the document with all sections, see nature.com/documents/nr-reporting-summary-flat.pdf

Life sciences study design

All studies must disclose on these points even when the disclosure is negative.

Sample size	n=6-10 in each experiments.
Data exclusions	Data was not exclusion from the experiment unless apparent failures
Replication	Each experiments were repeated more than 3 times to verify the reproducibility.
Randomization	In animal studies, we randomly allocated animals into groups such that as animals were added to the experiment, the numbers of animals in each group did not significantly differ.
Blinding	We needed to investigate the difference which had not been known between different groups. Thus we did not use blinding in the study.

Reporting for specific materials, systems and methods

We require information from authors about some types of materials, experimental systems and methods used in many studies. Here, indicate whether each material, system or method listed is relevant to your study. If you are not sure if a list item applies to your research, read the appropriate section before selecting a response.

Materials & experimental systems

n/a	Involved in the study	
☐	☒	Antibodies
☒	☐	Eukaryotic cell lines
☒	☐	Palaeontology
☐	☒	Animals and other organisms
☒	☐	Human research participants
☒	☐	Clinical data

Methods

n/a	Involved in the study	
☒	☐	ChIP-seq
☒	☐	Flow cytometry
☒	☐	MRI-based neuroimaging

Antibodies

Antibodies used

ZO-1 antibody (Rabbit, Life technology)
Occludin antibody (Rabbit, Life technology)
Claudin-2 antibody (Rabbit, Life technology)
Claudin-4 antibody (Rabbit, Life technology)
F4-80 antibody (Rat anti mouse, Bid-Rad)
NF-κB p-p65 antibody (Rabbit, Cell Signaling Technology)
NF-κB p65 antibody (Rabbit, Cell Signaling Technology)
CYP2E1 antibody (Rabbit, Abcam)
TLR4 antibody (Rabbit, Cell Signaling Technology)
MyD 88 antibody (Rabbit, Cell Signaling Technology)
TNF-α antibody (Rabbit, Cell Signaling Technology)

	GAPDH antibody (Rabbit, Cell Signaling Technology)
Validation	The validation of antibodies used for WB were performed by SDS-PAGE by the manufacturer, with relevant data presented on the manufacturer website.

Animals and other organisms

Policy information about studies involving animals; ARRIVE guidelines recommended for reporting animal research

Laboratory animals	C57BL/6J Mice
Wild animals	C57BL/6 mice, 10-12 week-old
Field-collected samples	This study did not involve field-collected samples.
Ethics oversight	All animal experiments were approved by the Animal Ethical and Welfare Committee of University of Macao (No. UM-ARE-015-2018).

Note that full information on the approval of the study protocol must also be provided in the manuscript.
