# Supplementary material for: Oxidation of fish oil exacerbates alcoholic liver disease by enhancing intestinal dysbiosis in mice
Source: Commun Biol. 2020 Sep 2;3:481. doi: 10.1038/s42003-020-01213-8 (PMC7468239; doi:10.1038/s42003-020-01213-8)

Supplementary Information

Supplementary Figure 1

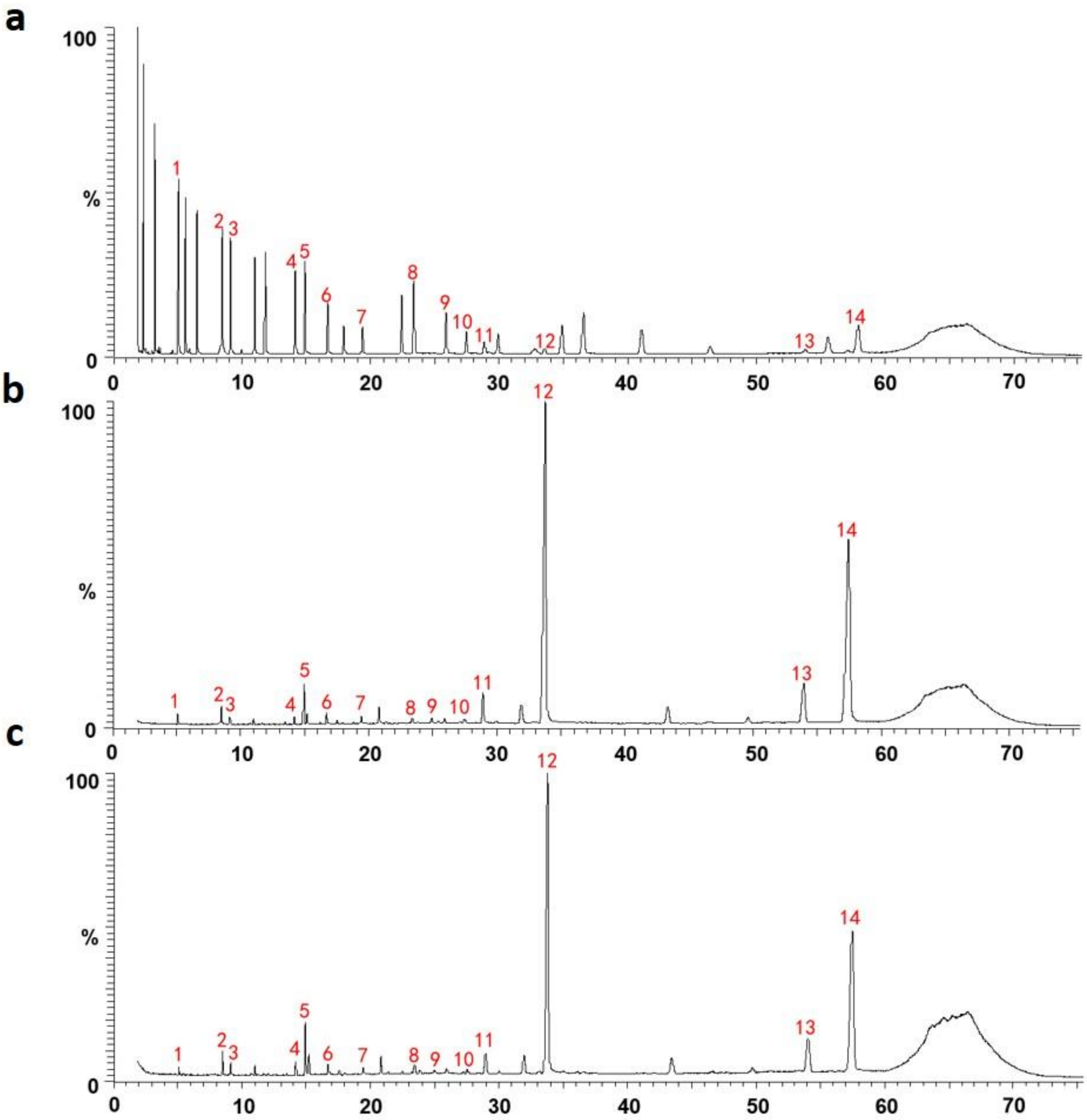

Supplementary Figure 1. Representative total ion chromatograms of the mixed standards. **a** unoxidized fish oil. **b** oxidized fish oil. **c** **1**, 14:0; **2**, 16:0; **3**, 16:1 n-7; **4**, 18:0; **5**, 18:1 n-9; **6,** 18:2 n-6; **7,** 18:3 n-3 (ALA); **8**, 20:1 n-9; **9**, 20:2 n-6; **10**, 20:3 n-6; **11**, 20:4 n-6 (AA); **12**, 20:5 n-3 (EPA); **13**, 22:5 n-3(DPA); **14**, 22:6 n-3 (DHA). Heptadecanoic acid (17:0) was used as an internal standard.

Supplementary Figure 2

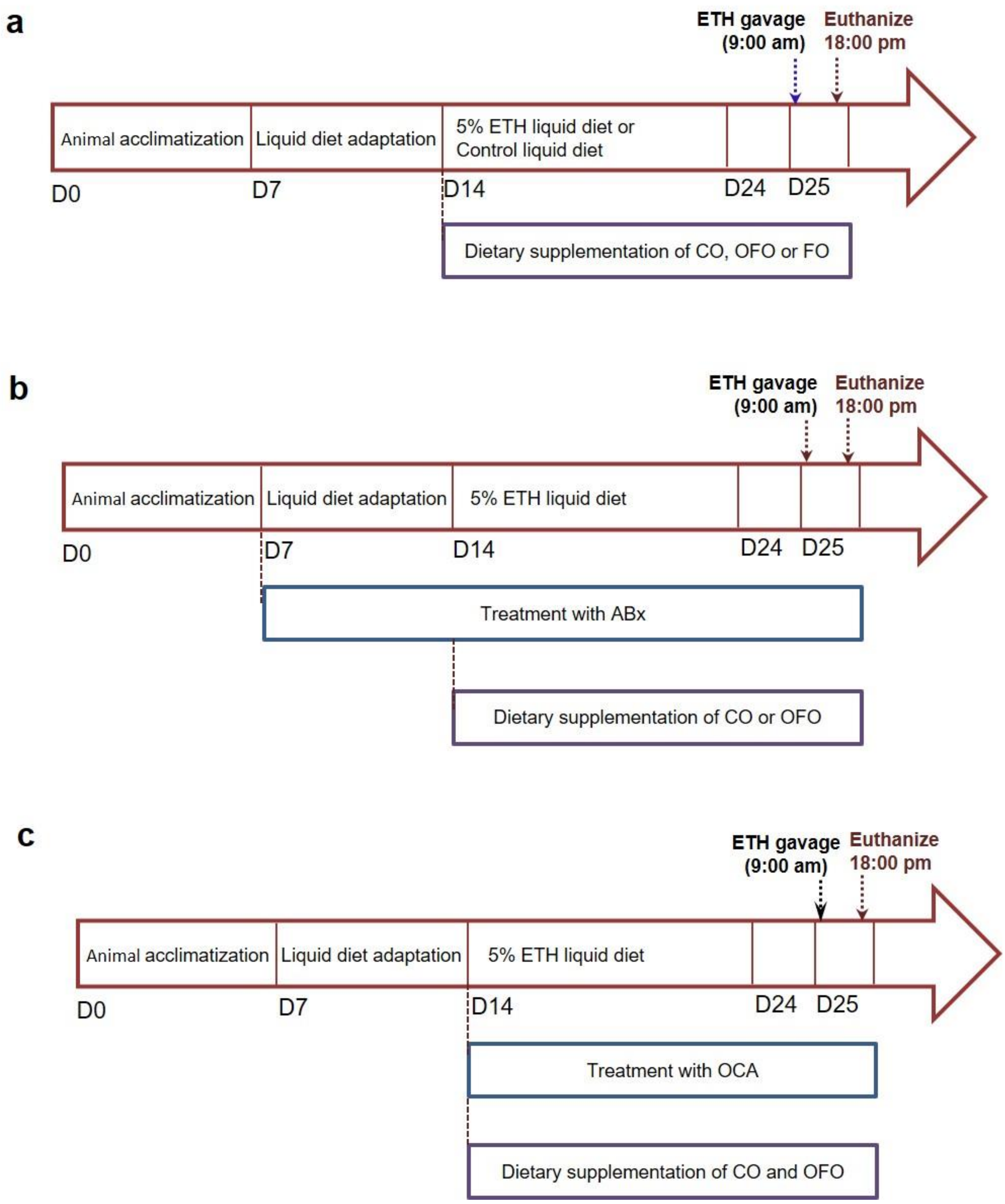

Supplementary Figure 2. Experimental schemes. **a** Ethanol-induced liver injury experiment by chronic- plus-single binge ethanol feeding. **b** ABx-treated experiment. **c** Obeticholic acid (OCA)- treatment experiment.

Supplementary Figure 3

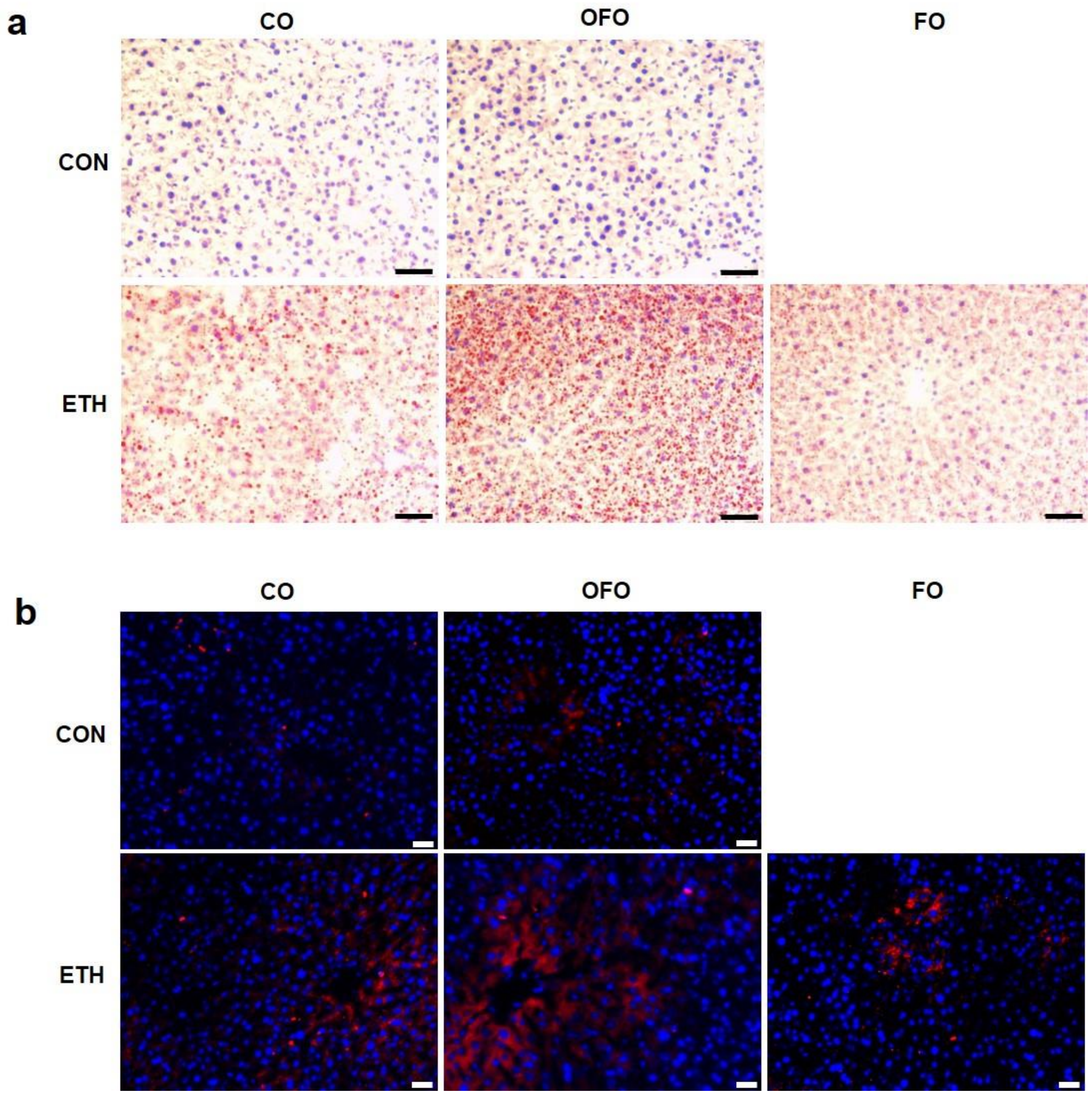

Supplementary Figure 3. Dietary OFO exacerbates hepatic lipid accumulation and macrophage infiltration induced by alcohol exposure. **a** Representative images of Red Oil O staining (scale bar, 200 μm). **b** F4/80 staining (scale bar, 25 μm).

Supplementary Figure 4

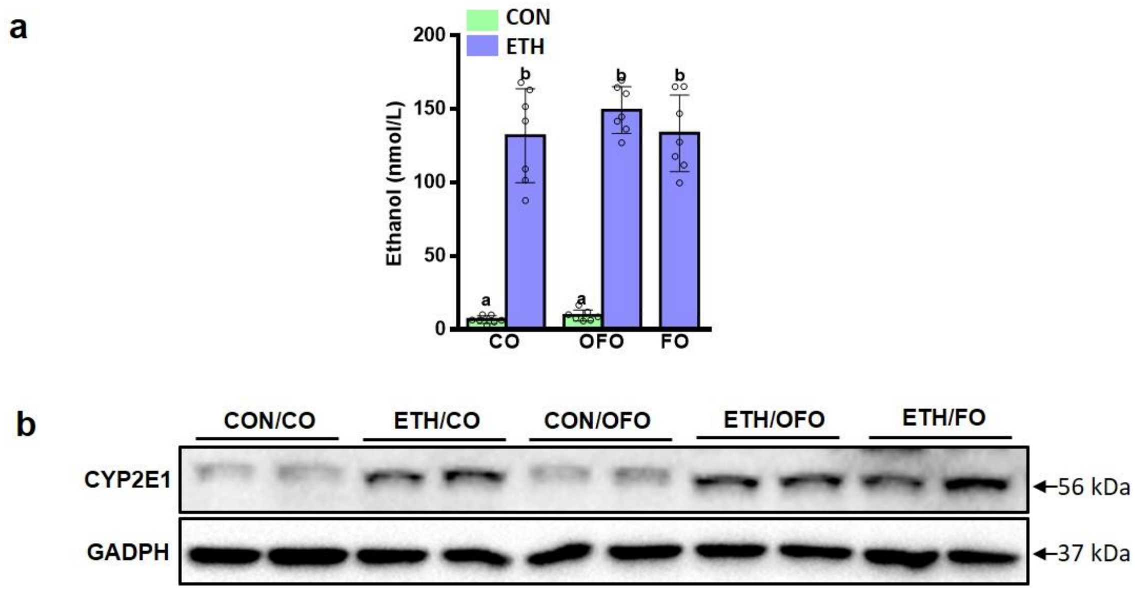

Supplementary Figure 4. Dietary OFO does not affect the absorption and metabolism of ethanol (n=7). **a** Plasma ethanol level. **b** the protein expression of CYP2E1 enzyme in the liver. Data were expressed as the mean ± SD.

Supplementary Figure 5

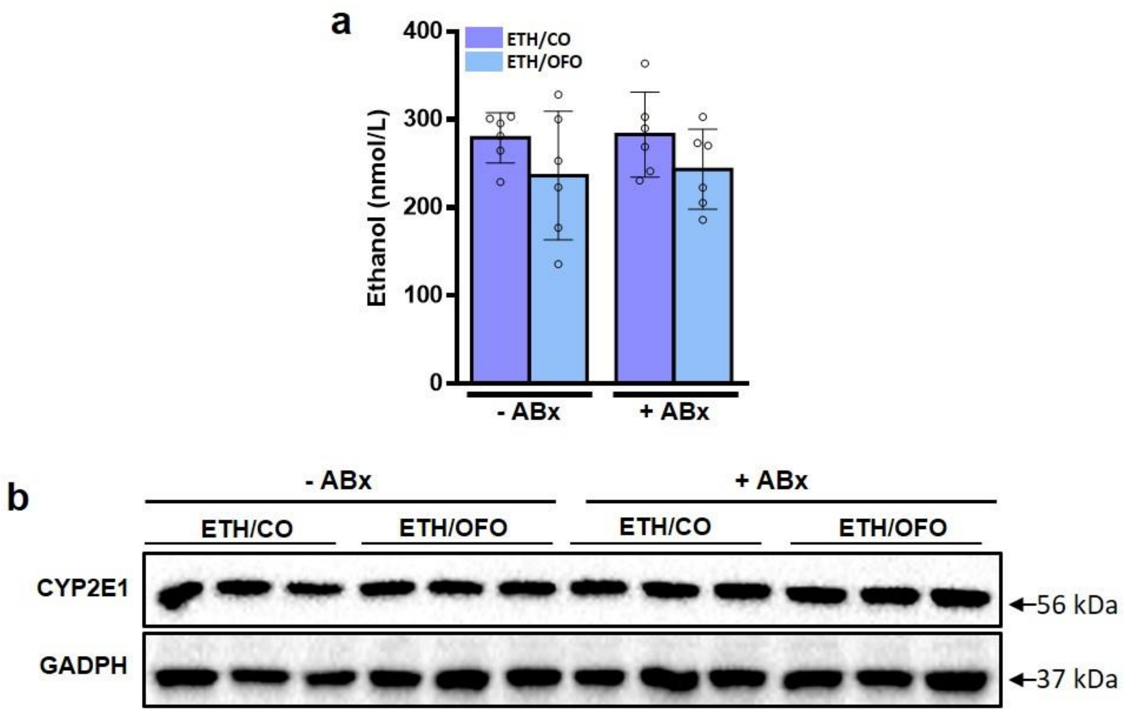

Supplementary Figure 5. Abx treatment does not affect the absorption and metabolism of ethanol. **a** Plasma ethanol level. **b** the protein expression of CYP2E1 enzyme. Data were expressed as the mean ± SD (n=6).

Supplementary Figure 6

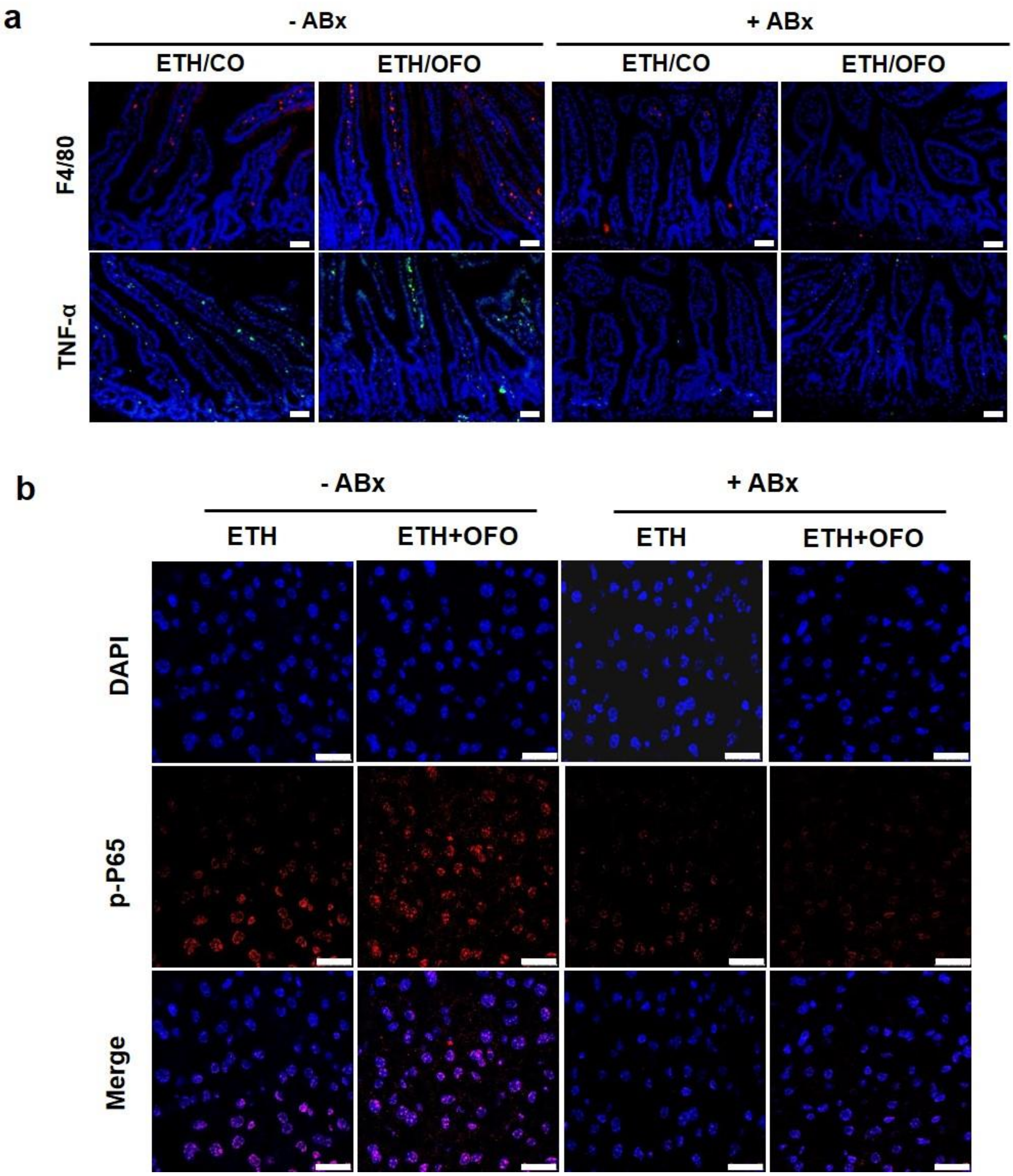

Supplementary Figure 6. Abx treatment abolishes OFO-aggravated intestinal and hepatic inflammation in ethanol-fed mice. **a** Immunofluorescent staining of intestinal TNF-α and F4/80 in jejunum tissues (scale bar, 25 μm). **b** p-p65 in the liver. Nucleus was stained with DAPI (blue; scale bar, 25 μm).

Supplementary Figure 7

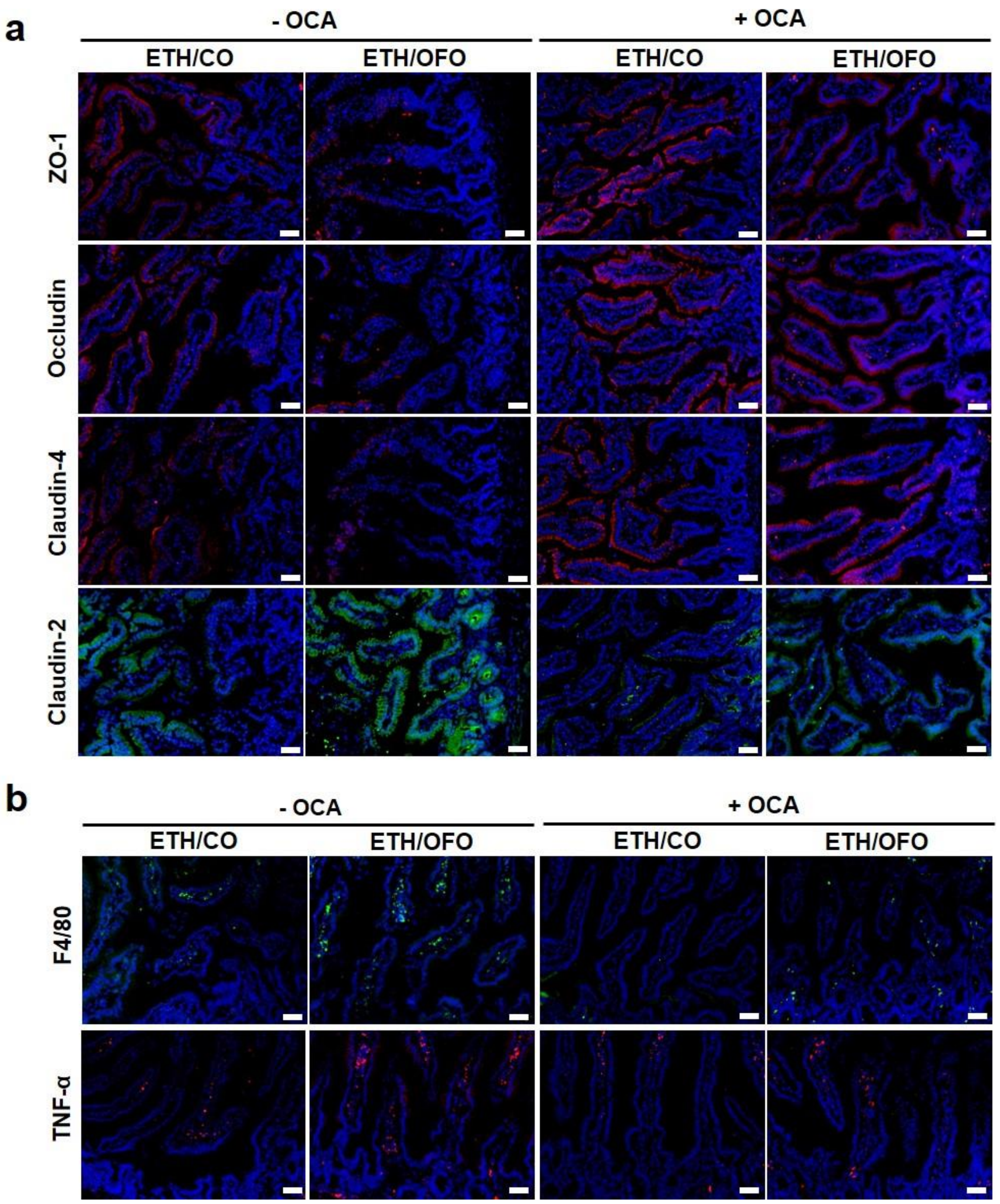

Supplementary Figure 7. Obeticholic acid (OCA) treatment reverses OFO-aggravated intestinal barrier dysfunction and intestinal inflammation in ethanol-fed mice. **a** Immunofluorescent staining of intestinal TJ proteins, including ZO-1, occluding, claudin-4, and claudin-2 in the sections of jejunum tissue (scale bar, 25 μm). **b** intestinal TNF-α and F4/80. Nucleus was stained with DAPI (blue; scale bar, 25 μm).

Supplementary Table 1. Fatty acid profiles of unoxidized fish oil and oxidized fish oil determined by GC-MS.

No.	Fatty acids		Unoxidized fish oil (μg/mg)	Oxidized fish oil (μg/mg)
	**Common name**	**Symbol**		
**1**	Myristic acid	14:0	1.53 ± 0.08	0.69 ± 0.09 ***
**2**	Palmitic acid	16:0	2.53 ± 1.63	2.96 ± 0.12
**3**	Palmitoleic acid	16:1 n-7	1.50 ± 0.13	1.73 ± 0.17
**4**	Stearic acid	18:0	1.35 ± 0.61	2.52 ± 0.12*
**5**	Oleic acid	18:1 n-9	11.11 ± 0.45	8.91 ± 0.80
**6**	linoleic acid	18:2 n-6	3.05 ± 0.20	1.97 ± 0.19**
**7**	α-Linolenic acid (ALA)	18:3 n-3	2.33 ± 0.10	1.26 ± 0.13***
**8**	Eicosenoic acid	20:1 n-9	1.73 ± 0.20	1.99 ± 0.23
**9**	Eicosadienoic acid	20:2 n-6	1.98 ± 0.12	0.99 ± 0.12***
**10**	Eicosatrienoic acid	20:3 n-6	1.95 ± 0.08	1.05 ± 0.08***
**11**	Arachidonic acid (AA)	20:4 n-6	15.80 ± 0.58	6.41 ± 0.58***
**12**	Eicosapentaenoic acid (EPA)	20:5 n-3	251.2 ± 11.2	123.2 ± 12.7***
**13**	Docosapentaenoic acid (DPA)	22:5 n-3	36.51 ± 1.51	18.39 ± 1.97***
**14**	Docosahexaenoic acid (DHA)	22:6 n-3	202.9 ± 8.8	83.71 ± 10.24***

Supplementary Table 2. Compositions of the modified Lieber-DeCarli liquid diets[a].

**Ingredients** (g/L)	**CON/CO**	**ETH/CO**	**CON/OFO**	**ETH/OFO**	**ETH/FO**
Casein	41.4	41.4	41.4	41.4	41.4
L-cystine	0.50	0.50	0.50	0.50	0.50
D, L-Methionine	0.30	0.30	0.30	0.30	0.30
Cellulose	10.0	10.0	10.0	10.0	10.0
Maltose Dextrin	115	44.8	115	44.8	44.8
Corn Oil	39.6	39.6	19.8	19.8	19.8
Oxidized fish oil	-	-	19.8	19.8	-
Fish oil [b]	-	-	-	-	19.8
Mineral Mix	8.8	8.8	8.8	8.8	8.8
Vitamin Mix	2.5	2.5	2.5	2.5	2.5
Choline Bitartrate	0.53	0.53	0.53	0.53	0.53
Vitamin E Acetate	0.20	0.20	0.20	0.20	0.20
95% Ethanol (*v*/*v*), mL/L	-	52.6	-	52.6	52.6

[a] Mice in alcohol groups were fed the modified Lieber-DeCarli alcohol liquid diets (TROPHIC Animal Feed High-tech Co., Ltd. Nantong, Jiangsu, China) with an energy composition of 18% protein, 19% carbohydrate, 35% fat and 28% ethanol, and mice in control groups were fed the Lieber-DeCarli control diet, in which ethanol was isocalorically replaced by maltose-dextrin.

[b] FO is composed of 45.5% EPA, 35.5% DHA and 13.0% other omega-3 PUFA.

Supplementary Table 3. Primary antibodies used in immunoblot analysis.

Primary antibody	Full name	Dilution	Company
CYP2E1	Cytochrome P4502E1	1 : 1000	Abcam
Claudin-2	Claudin-2	1 : 1000	Invitrogen
Claudin-4	Claudin-4	1 : 1000	Invitrogen
MyD88	Myeloid differentiation primary response 88	1 : 1000	Cell Signaling
Occludin	Occludin	1 : 1000	Invitrogen
TLR4	Toll-like receptor-4	1 : 1000	Cell Signaling
p65	Transcription factor p65	1 : 1000	Cell Signaling
p-p65	Phosphorylated transcription factor p65	1 : 1000	Cell Signaling
ZO-1	Zonula occluding-1	1 : 1000	Invitrogen

Original western blots in Figure 2c and Figure 3b

Figure 2c

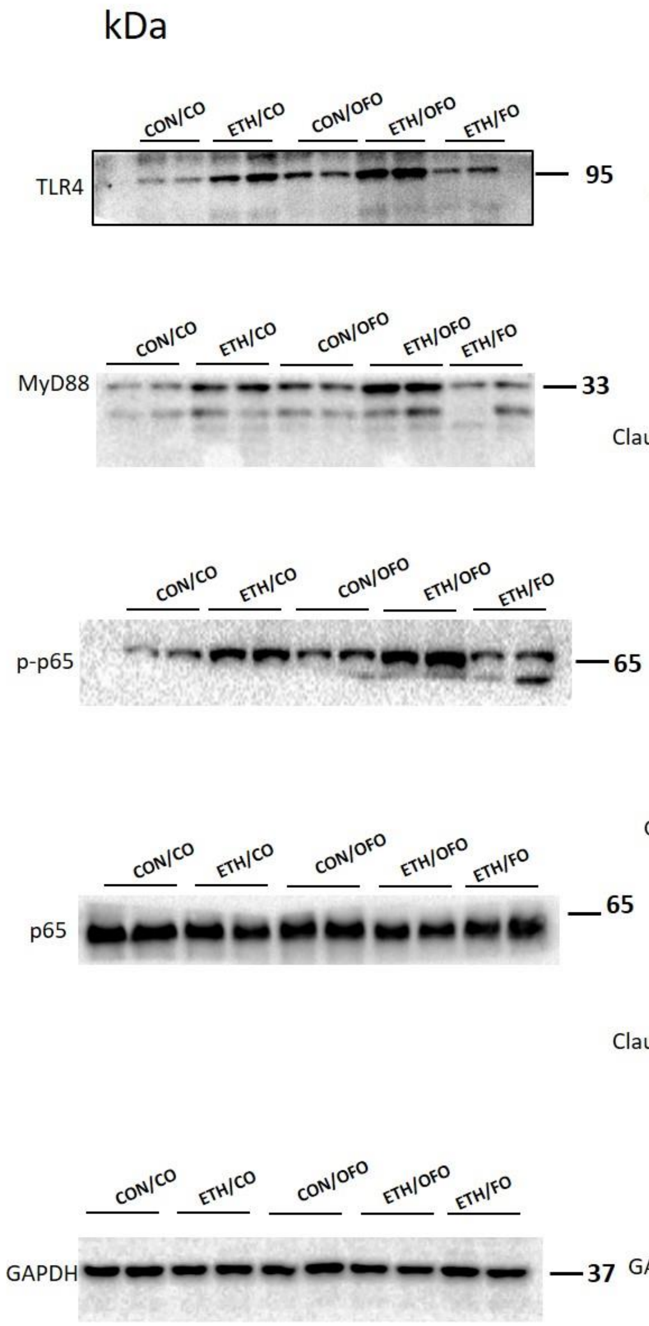

Figure 3b

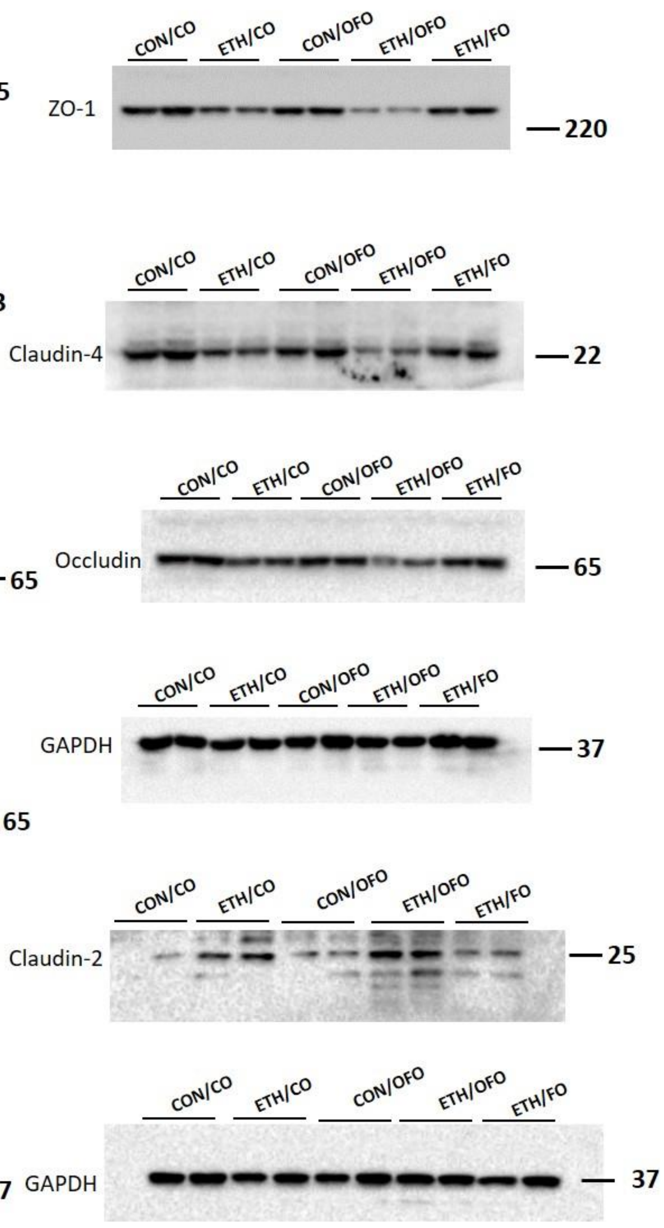

Original western blots in Supplementary Figure 4b and Figure 5b

Supplemental Figure 4b

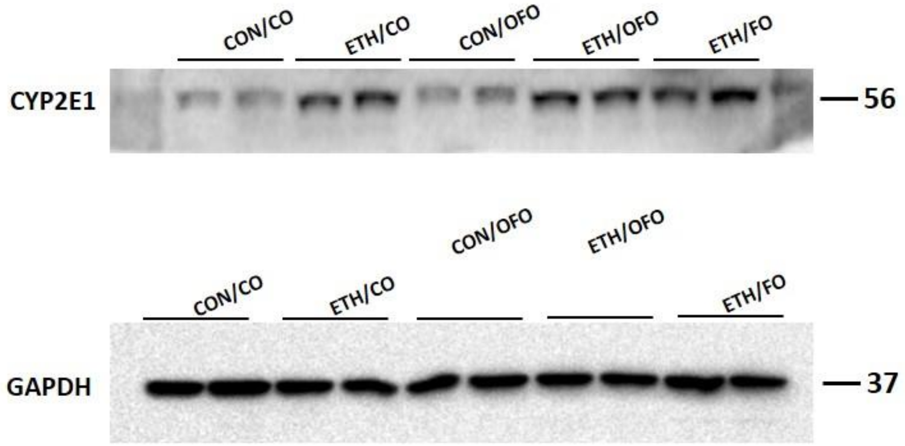

Supplemental Figure 5b

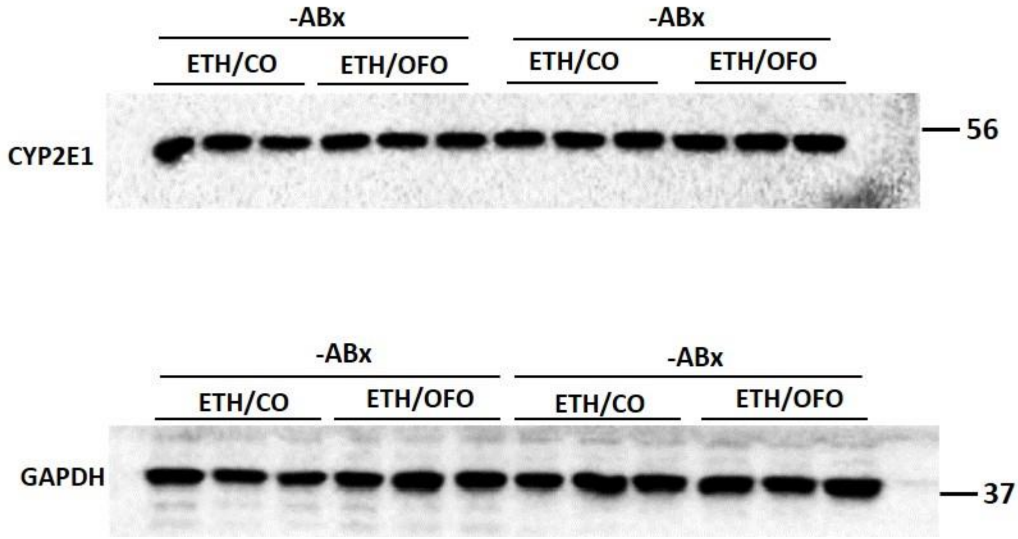

Original western blots in Figure 5e and Figure 6d

Figure 5e

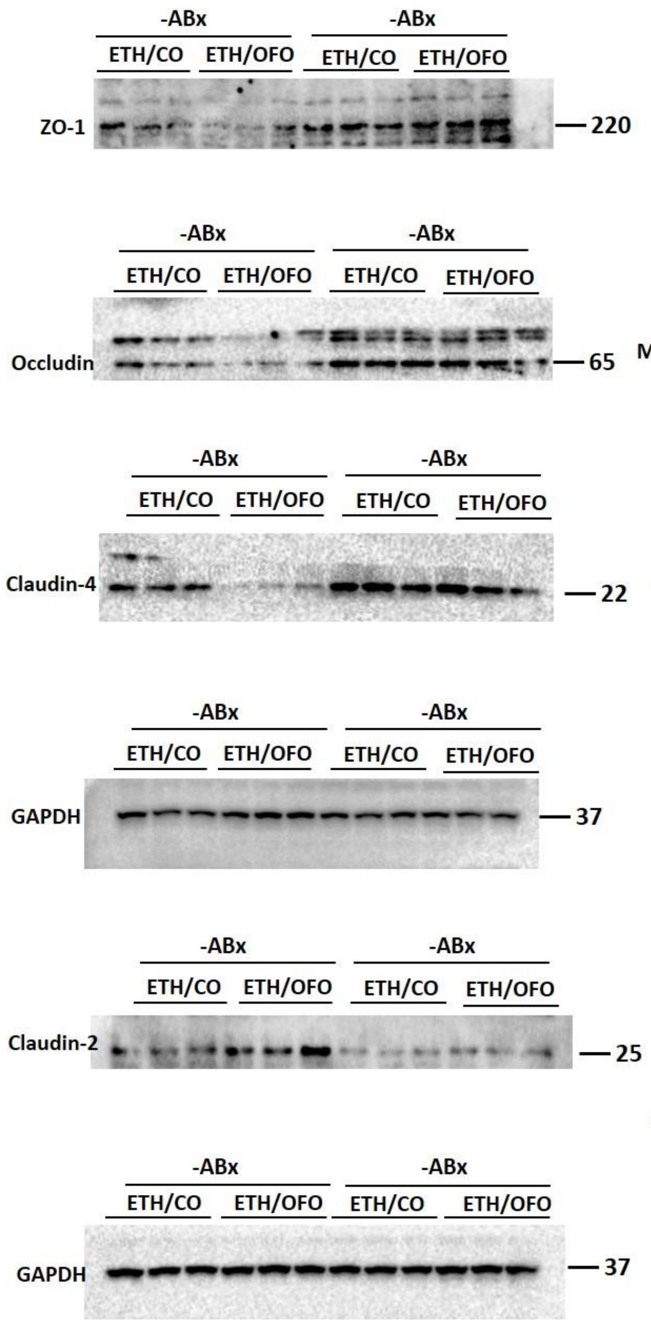

Figure 6d

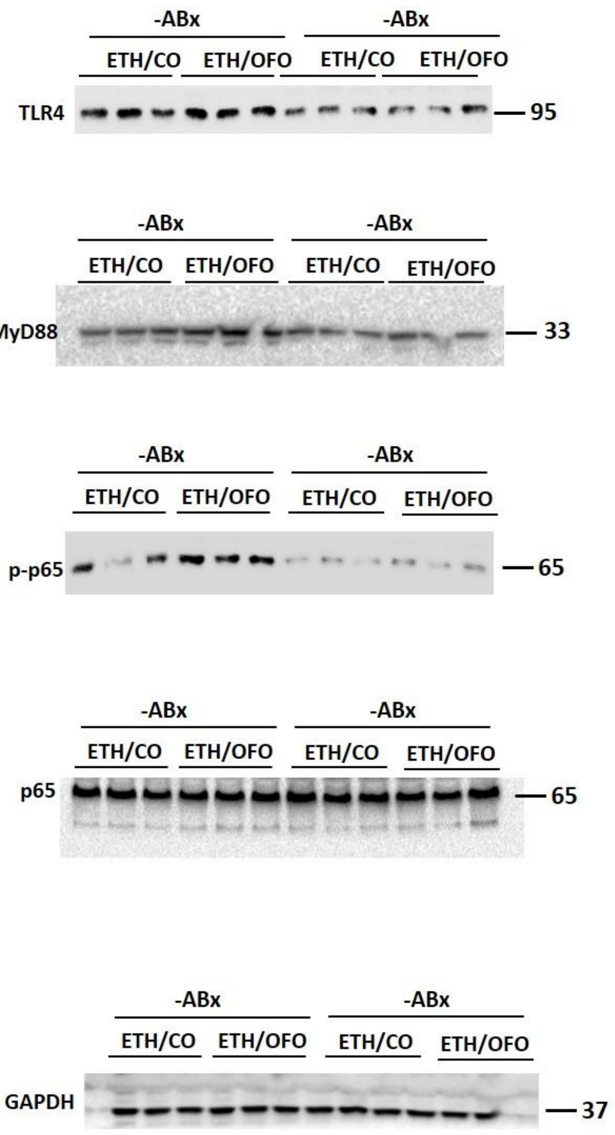

Supplement: Supplementary file 2 — Supplementary Information [file 42003_2020_1213_MOESM2_ESM.pdf]
