# Supplementary material for: Oxidation of fish oil exacerbates alcoholic liver disease by enhancing intestinal dysbiosis in mice
Source: Commun Biol. 2020 Sep 2;3:481. doi: 10.1038/s42003-020-01213-8 (PMC7468239; doi:10.1038/s42003-020-01213-8)
Supplement: Supplementary file 3 — Description of additional supplementary files [file 42003_2020_1213_MOESM3_ESM.docx]

Description of Additional Supplementary Files

Supplementary Data 1: Source data underlying plots shown in figures.
